# Evaluating the Efficacy and Accuracy of AI-Assisted Diagnostic Techniques in Endometrial Carcinoma: A Systematic Review

**DOI:** 10.7759/cureus.60973

**Published:** 2024-05-24

**Authors:** Jawaria Changhez, Simran James, Fazilat Jamala, Shandana Khan, Muhammad Zarak Khan, Sana Gul, Irta Zainab

**Affiliations:** 1 Gynecology, Kabir Medical College, Peshawar, PAK; 2 Gynecology, Rehman Medical Institute, Peshawar, PAK; 3 Obstetrics and Gynecology, Northwest General Hospital and Research Center, Peshawar, PAK; 4 General Surgery, Medical Teaching Institution (MTI) - Hayatabad Medical Complex, Peshawar, PAK; 5 Community Medicine, Pak International Medical College, Peshawar, PAK; 6 Gynecology, Medicsi Hospital, Islamabad, PAK

**Keywords:** ai and robotics in healthcare, neoplasm staging, diagnostic imaging, artificial intelligence oncology, endometrial carcinoma

## Abstract

Diagnosing endometrial carcinoma correctly is essential for appropriate treatment, as it is a major health risk. As machine learning (ML) and artificial intelligence (AI) have grown in popularity, so has interest in their potential to improve cancer diagnosis accuracy. In the context of endometrial cancer, this study attempts to examine the efficacy as well as the accuracy of AI-assisted diagnostic approaches. Additionally, it aims to methodically evaluate the contribution of AI and ML techniques to the improvement of endometrial cancer diagnosis. Following PRISMA guidelines, we performed a thorough search of numerous databases, including Medline via Ovid, PubMed, Scopus, Web of Science, and Google Scholar. Ten years were searched, encompassing both basic and advanced research. Peer-reviewed papers and original research studies that explicitly looked at the application of AI/ML in endometrial cancer diagnosis were the main targets of the well-defined selection criteria. Using the Critical Appraisal Skills Programme (CASP) methodology, two independent researchers conducted a thorough screening process and quality assessment of included studies. The review found a notable inclination towards the effective use of AI in endometrial carcinoma diagnostics, namely in the identification and categorization of endometrial cancer. Artificial intelligence models, particularly Convolutional Neural Networks (CNNs) and deep learning algorithms have shown remarkable precision in detecting endometrial cancer. They frequently achieve or even exceed the diagnostic proficiency of human specialists. The use of artificial intelligence in medical diagnostics signifies revolutionary progress in the field of oncology. AI-assisted diagnostic tools have demonstrated the potential to improve the precision and effectiveness of cancer diagnosis, namely in cases of endometrial carcinoma. This innovation not only enhances the quality of patient care but also indicates a transition towards more individualized and efficient treatment approaches in the field of oncology. The advancement of AI technology is expected to play a crucial role in medical diagnostics, particularly in the field of cancer detection and treatment, perhaps leading to a significant transformation in the approach to these areas.

## Introduction and background

Endometrial carcinoma (EC) is a typical kind of gynecological cancer with a wide range of clinical symptoms that make diagnosis very difficult [1]. Endometrial cancer is the most common gynecologic malignancy in the U.S. and is second only to cervical cancer worldwide. The World Health Organisation (WHO) categorizes endometrial carcinoma into histological categories [1]. Recent data show that endometrial cancer incidence has been rising in both industrialized and developing nations, partially as a result of changing lifestyle choices and longer life expectancies [1]. Introducing artificial intelligence (AI) into medical diagnostics has created new opportunities to improve the precision and effectiveness of cancer identification and characterization [2]. Artificial intelligence (AI) has transformed medical imaging and diagnostics, especially with machine learning and deep learning [2]. The accuracy of diagnosis of many malignancies, including endometrial carcinoma, has improved because of these technologies [2]. Artificial intelligence (AI) algorithms can evaluate complicated medical imaging data, including ultrasound, CT, and MRI scans, to help with early cancer detection and correct diagnosis [3]. The potential of AI-assisted diagnostic tools to improve conventional diagnostic procedures in the setting of endometrial cancer has been investigated [3]. According to Feng et al. [4], these methods include imaging data analysis to anticipate tumor features before surgery, distinguish benign from malignant lesions, and enhance the precision of disease staging and grading. Enhancement of image processing and classification is part of the integration of AI in medical imaging, especially in endometrial cancer, since this is essential for precise diagnosis and treatment planning [5]. Especially in the realm of cancer, integrating AI in medical diagnostics has been a revolutionary advance. Extensive dataset processing and analysis capabilities of AI have been beneficial in spotting trends and abnormalities that might be signs of cancers such as endometrial carcinoma [2]. For example, machine learning models have been used to analyze histopathology pictures, providing diagnostic accuracy that exceeds conventional approaches [4].

The diversity of endometrial cancer calls for a sophisticated diagnostic process in which artificial intelligence may be beneficial. Even for seasoned pathologists, distinguishing between distinct histological subtypes of endometrial cancer may be difficult, but AI systems can do so [1]. Since the prognosis and course of therapy for each subtype might differ significantly, this separation is essential. For instance, endometrioid adenocarcinomas often have a better prognosis than serous carcinomas, accounting for fewer patients [1].

Owing to the increasing number of EC cases worldwide and difficulties related to its diagnosis and prognosis, the application of artificial intelligence (AI) in pathology has recently become one of the most noticeable issues [1]. In these last couple of years, the growth of AI, especially machine learning (ML) and clinical decision support systems (CDSSs), has provided exciting directions for improving diagnostic accuracy and clinical acts in EC management. AI models like those offered by Wu et al. [3] show the possibility of ML algorithm's accuracy at predicting the risk of EC with a view to prognostic purposes. Employing machine learning tools, AI models can determine whether it is a type I or type II histology, the stage, and the grade of EC through pre-operational examinations and clinical data. These models will become the building blocks of an evolving and complex CDSS, which will be used for rapid and accurate diagnosis to improve patient outcomes and operational efficiency of the delivery system.

Moreover, there have also been notable developments in the use of AI in medical imaging for endometrial cancer. Artificial intelligence (AI) algorithms have been created to evaluate MRI and ultrasound data, offering valuable insights into tumor features such as size, lymph node involvement, and depth of myometrial invasion [3]. The staging of the illness, which directly affects therapy choices, depends on these observations. AI was used in research to evaluate MRI images, and the models could predict deep myometrial invasion, which is crucial for staging endometrial cancer, with an accuracy rate of over 90% [6]. AI influences prognosis prediction and therapy planning in addition to diagnosis. Treatment strategies may now be more individually tailored thanks to machine learning algorithms that anticipate patient outcomes based on imaging and clinical data [7]. For example, AI systems can forecast the chance of metastasis or recurrence, essential information for choosing how aggressively to treat the disease [8].

The use of AI for endometrial cancer has been made possible mainly by its accuracy in medical imaging. In conjunction with advanced imaging methods, AI algorithms have significantly improved the identification and characterization of endometrial lesions. In contrast to conventional imaging analysis techniques, deep learning models used for MRI scans have shown a sensitivity of up to 92% in diagnosing endometrial malignancy [9]. Improved patient survival rates are directly linked to early identification, which is made possible by this high degree of sensitivity. AI has been crucial in improving the efficiency of medical imaging analysis and diagnosis accuracy. AI-powered devices can process and analyze medical pictures more quickly than human radiologists, which shortens the time it takes to diagnose a patient and allows for a speedier start to therapy [10]. The capacity to digest information quickly is beneficial in high-volume clinical settings where prompt diagnosis may have a significant influence on patient outcomes.

AI plays a part in improving medical imaging by improving both picture quality and interpretability. Methods like machine learning and deep learning-based image augmentation and enhancement have been developed to increase the clarity and depth of medical pictures and help with more precise diagnosis and treatment planning [11]. A significant problem in the diagnosis of endometrial cancer is the yielding of confusing or unclear data from standard imaging modalities. In these circumstances, enhanced picture quality is very crucial. AI has also shown promise in forecasting the prognosis and responsiveness to therapy for patients with endometrial cancer. Artificial intelligence (AI) models can forecast therapy effectiveness, possible adverse effects, and overall prognosis by evaluating a mix of clinical, pathological, and imaging data [5]. This predictive skill is crucial in personalized medicine, where treatment regimens may be customized to each patient's unique profile, maximizing effectiveness and reducing needless treatments.

Despite the notable progress made in AI-assisted diagnostics for endometrial cancer, there exists a need for thorough and multi-modal assessments of these technologies. Most research endeavors tend to concentrate on isolated elements, such as the analysis of images or the interpretation of histological data, without effectively integrating these factors with clinical results and the demographic characteristics of patients. Furthermore, a dearth of longitudinal investigations exists that evaluate the enduring effectiveness and precision of artificial intelligence (AI) technologies inside authentic clinical environments. The lack of knowledge in this area impedes the comprehensive comprehension of artificial intelligence's complete capabilities and constraints in endometrial cancer diagnostics. This is especially true when considering the broad range of patient populations and various phases of the illness.

The main focus of this systematic review is to address the fragmented and diverse nature of research about AI-assisted diagnostic tools in the context of endometrial cancer. Previous research has yielded a range of results characterized by differences in the quality of methodology, breadth of the investigation, and relevance to clinical practice. Fragmentation hinders healthcare practitioners from developing a unified comprehension of the efficacy and dependability of artificial intelligence (AI) solutions within this domain. There is a need for a comprehensive and methodical synthesis and examination of the existing body of research to ascertain recurring patterns, identify areas of limited understanding, and detect any possible biases. This endeavor will contribute to a more precise understanding of the present status of artificial intelligence in endometrial cancer diagnosis.

The rationale for conducting this systematic review arises from the increasing intricacy and potential of artificial intelligence (AI) in the domain of cancer diagnostics, with a specific focus on endometrial carcinoma. In light of the swift advancements in artificial intelligence (AI) technologies and their diverse implementations in the realms of medical records, including past medical conditions, laboratory test results, imaging reports, treatments received, and other relevant clinical data, there arises a pressing need to consolidate extant scholarly investigations to grasp their practical effectiveness and inherent constraints comprehensively. This review aims to collect and assess the current studies to guide future research, improve clinical practice, and enable the invention of more efficient AI-assisted diagnostic tools for endometrial cancer, examining and consolidating existing research on endometrial cancer detection techniques helped by artificial intelligence (AI). The accuracy and efficacy of various methods in a medical context are assessed.

## Review

Materials and methods

Search Strategy

This systematic review assesses the efficiency and accuracy of AI-assisted diagnostic approaches in endometrial cancer. The approach has been developed to guarantee a thorough and impartial compilation, examination, and integration of relevant literature while conforming to the principles outlined in the Preferred Reporting Items for Systematic Reviews and Meta-Analyses (PRISMA). A comprehensive investigation was carried out across many databases, such as Medline via Ovid, PubMed, Scopus, Web of Science, and Google Scholar, to include a wide range of scholarly literature. The search included the last ten years' duration of each database from 2013 to 2023. The decision to use a broad period was made to include both fundamental and advanced research, offering a comprehensive perspective on the development of the discipline. Due to the nature of this systematic review, which involves synthesizing previously published research, the acquisition of Research Ethics Committee permission was considered unnecessary. The present evaluation lacks the inclusion of novel investigations involving human subjects or animals conducted by any of the authors.

Eligibility Criteria

Table 1 shows the inclusion and exclusion criteria for the most relevant articles. The search strategy was made with great attention. Choosing and arranging keywords and phrases were done carefully, with Boolean operators included to increase search precision. Various synonyms and variations of terms related to AI, ML, endometrial carcinoma, endometrial cancer, diagnostics, and imaging were included in the thesaurus. Here, we set out to gather all the studies that have looked at endometrial cancer detection using AI and ML. Research into the use of various diagnostic tools, such as histology analysis and advanced imaging techniques, falls under this category. Exporting the recognized articles to EndNote allowed for more efficient management. The first step in the screening process was to remove any duplicate entries and irrelevant research. It was crucial to include this step so that we could focus on the most relevant articles and make the evaluation process easier.

**Table 1:** The inclusion and exclusion criteria.

Criteria	Inclusion	Exclusion
Publication Year	Papers published between 2013 and 2023.	Papers published before 2013.
Study Type	Peer-reviewed articles, systematic reviews, meta-analyses, and original research studies.	Editorials, commentaries, opinions, and non-peer-reviewed articles.
Subject	Studies focusing on AI and ML techniques in the diagnosis of endometrial carcinoma.	Studies not specifically addressing AI/ML in endometrial carcinoma diagnosis.
Language	Articles published in English.	Articles published in languages other than English.
Data Availability	Studies with accessible and clear data/results.	Studies with inaccessible data or unclear results.
Methodology	Studies employing AI/ML for diagnostic imaging, histopathological analysis, or other diagnostic tools.	Studies focusing on treatment, prognosis, or other aspects not related to diagnosis.
Outcome Measures	Studies providing specific diagnostic outcomes (e.g., sensitivity, specificity, accuracy).	Studies lacking clear diagnostic outcome measures.

Data Extraction

Researchers worked independently to maintain impartiality and minimize biases during the screening process. The procedure included an initial examination of titles and abstracts, followed by a comprehensive evaluation of the whole texts of the papers that were selected as finalists. The first stage of the research endeavor focused on expeditiously identifying publications that possibly aligned with the overarching goals of the study. The subsequent stage included comprehensively scrutinizing the whole text to validate its pertinence. The provided document contains crucial details, including the name of the primary author, the year of publication, the place of origin, the field of application, the research design, the number of databases that were searched, the number of studies that were included, and the significant results or conclusions. Furthermore, any limits or obstacles identified in these studies were documented. The use of a systematic methodology for data extraction guaranteed the attainment of both consistency and thoroughness in capturing information for further analysis. The papers included in this analysis were subjected to a thorough evaluation of their quality using the Critical Appraisal Skills Programme (CASP) tool. This methodology enabled a thorough assessment of the methodological rigor, dependability, and validity of the included studies. Quality evaluation played a crucial role in evaluating the robustness of the research evidence and detecting any possible biases or methodological limitations. The data from the studies included in this review were synthesized and analyzed to answer the study goals effectively. The process included classifying the research according to their areas of interest, methodology used, and principal conclusions. This synthesis aimed to discern prevalent themes, patterns, and deficiencies within the existing body of research concerning the use of artificial intelligence in the diagnosis of endometrial cancer. The study also included a comprehensive evaluation of the procedures used in the studies, the strength of their conclusions, and the ramifications of these findings for both clinical application and future scholarly investigations. During the evaluation process, ethical issues were diligently followed, with a special emphasis on upholding the intellectual rights of the original study authors. All sources were acknowledged, and no proprietary or sensitive material was used without proper authorization.

Data Analysis and Synthesis

The authors analyzed and synthesized the results using a narrative text approach focused on using artificial intelligence to diagnose endometrial cancer.

Results

Study Selection Process

**Figure 1:**
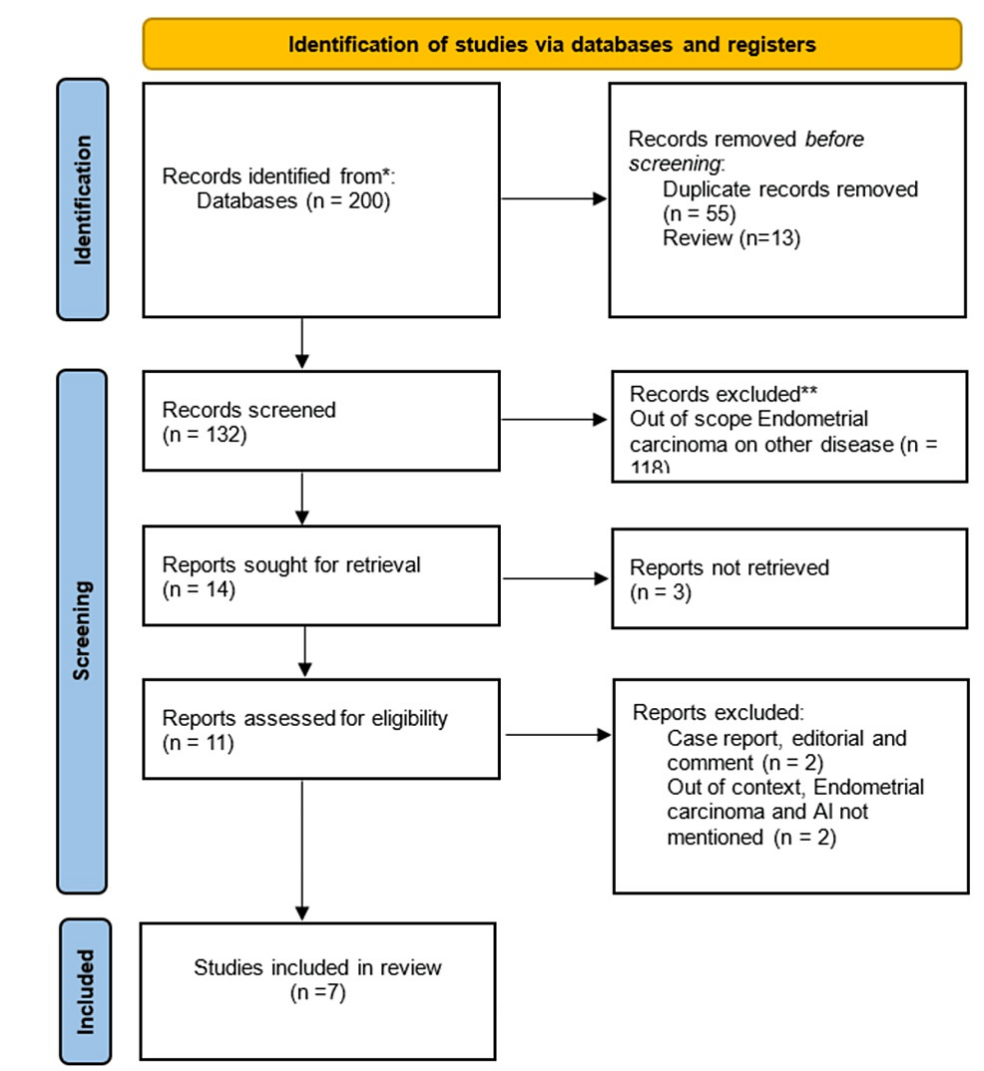
PRISMA showing the screening process and reasons for exclusion.

The initial database search retrieved 200 records. Out of these, 55 were excluded based on duplicates. The remaining 132 articles were screened for their title/abstract, and 14 publications were deemed for full-text screening. Out of which, three publications were excluded as their full text was not accessible. The full-text screening of the remaining 11 articles retrieved 7 to be included in the review. The detailed screening process and reason for exclusion is illustrated in PRISMA Figure 1.

Characteristics of Selected Studies

Table 2 shows the characteristics of the seven studies included in this review. All the articles included in this study were of good quality, considering the content related to the objectives of this study. All of them thoroughly explored the objective of the study.

**Table 2:** The characteristics of the studies included.

Study Reference	Year	Location	Study Design	Sample Size	AI Tool Used	Type of AI	Data Source
Erdemoglu et al. [12]	2023	Turkey	Observational Study	564	Various ML Models	Machine Learning	Clinical Data
Fell et al., [13]	2023	United Kingdom	Not Specified	2909	Whole slide images (WSI)	CNN	Pathological Data
Takahashi et al. [14]	2021	Not Specified	Analysis of hysteroscopic video data	177	Xception, MobileNetV2, EfficientNetB0	Deep Neural Networks	Hysteroscopic videos of the uterine lumen
Urushibara et al. [15]	2022	Japan	Retrospective study	388	Xception model on Deep Station Entry with GeForce RTX 2080Ti, Core i7-8700	Convolutional Neural Networks (CNN)	MRI scans from patients with endometrial cancer or non-cancerous lesions
Fremond et al. [16]	2023	Netherlands, UK, France, Italy, Canada, Australia, New Zealand	Combined analysis of randomized trials and clinical cohorts	2028	im4MEC	Deep Learning (Self-Supervised Learning, Attention Mechanism)	Haematoxylin and eosin-stained slides, molecular and clinicopathological data
Hart et al. [17]	2020	Not Specified	Population-based machine learning model development and comparison study	78,215	Logistic Regression, Neural Network, Support Vector Machine, Decision Tree, Random Forest, Linear Discriminant Analysis, Naive Bayes	Machine Learning	Ovarian Cancer Screening Trial (PLCO) dataset
Guerriero et al. [18]	2021		Comparative study of ML models using ultrasound data	333	k-NN, Naive Bayes, Neural Networks, SVM, Decision Tree, Random Forest, Logistic Regression	Machine Learning	Not Specified

Table 3 shows the diagnostic efficiency and accuracy of the seven studies included in the review.

**Table 3:** The diagnostic efficiency and accuracy. PPV: positive predictive value; NPV: negative predictive value

Study Reference	Algorithm Accuracy (PPV)	Diagnostic Efficiency	Sensitivity	Error Rate	Outcomes	
Erdemoglu et al. [12]	0.94	High	NA	6%	AI effectively predicted endometrial cancer risks; age, BMI, and endometrial thickness were key predictors.	
Fell et al. [13]	0.91	High	NA	8.9%	A fully supervised convolutional neural network (CNN) model was used in the research to categorize endometrial biopsy whole slide images (WSI) into "malignant," "other or benign," or "insufficient," with an overall accuracy of 91.1%.	
Takahashi et al. [14]	0.9	High	91.66%	9.71%	Combining deep neural network models yielded excellent endometrial cancer detection accuracy. This is noteworthy due to the limited sample size and illness complexity.	
Urushibara et al. [15]	AUC 0.88–0.95 for single and combined image sets	High	NA	NA	Convolutional neural networks (CNNs) exhibited exceptional diagnostic accuracy in identifying endometrial cancer through magnetic resonance imaging (MRI). Including additional types of photos enhanced the diagnosis accuracy for certain individual image collections.	
Fremond et al. [16]	Macro and class-wise AUROC: 0.874-0.876	High	NA	NA	im4MEC showed high performance in classifying endometrial cancer into molecular subgroups and identifying morpho-molecular correlates. It also contributed to prognostic refinement.	
Hart et al., [17]	Testing AUC of 0.96 (Random Forest), 0.91 (Neural Network)	High	Optimized using cross-validation for sensitivity, specificity, PPV, and NPV	Not explicitly stated	Random Forest and Neural Network models outperformed traditional risk models and human experts in identifying high-risk endometrial cancer cases with lower false positive rates.	
Guerriero et al. [18]	Varied (0.69 - 0.75)	High	Varied (0.66 - 0.84)	Not explicitly stated	AI models demonstrated comparable accuracy to the logistic model in detecting rectosigmoid endometriosis. Neural Network showed the best performance among the models with an accuracy of 0.73.	

Discussion

As mentioned in Table 3, in the study of Fell et al. [13], they utilized a fully supervised convolutional neural network (CNN) to classify endometrial biopsy WSIs as either cancerous, benign, or inadequate. These AI systems can prioritize pathologist review, which might lead to faster cancer diagnoses and better patient outcomes [13]. The degree of accuracy is considerable. This research shows that AI has great promise for improving pathology diagnosis accuracy, particularly when identifying malignant from benign samples or when there aren't enough samples for endometrial cancer biopsies. Takahashi et al. [14] demonstrated a remarkable overall accuracy of 0.94 in the diagnosis of endometrial cancer by employing a combination of deep neural network models. The AI models demonstrated a remarkable level of precision by accurately detecting the existence of endometrial cancer in most instances. This highlights the potential of AI tools to improve diagnostic accuracy in medical contexts. According to Takahashi et al. [14], using artificial intelligence in this investigation demonstrates a notable degree of diagnostic efficacy. The efficiency may be deduced from the high accuracy rate, indicating that the AI models can decrease the time and resources required for diagnosis compared to traditional methods.

A study by Urushibara et al. [15] has shown that convolutional neural networks (CNNs) have a high level of precision in detecting endometrial cancer through MRI, with an Area Under Curve (AUC) ranging from 0.88 to 0.95. The high level of precision displayed by the CNNs demonstrates their substantial capacity to differentiate between malignant and non-cancerous diseases, closely matching the diagnostic proficiency of expert radiologists. A high level of accuracy is vital in medical diagnostics since it guarantees a dependable identification of the condition, which is necessary for prompt and suitable patient treatment. The study by Urushibara et al. [15] emphasizes the capacity of CNNs to accelerate the diagnostic procedure, particularly in terms of efficiency. CNNs can expedite the diagnostic process by automating the interpretation of MRI scans, resulting in a substantial reduction in diagnosis time compared to conventional approaches that mainly depend on manual analysis by radiologists. The efficiency of this system does not sacrifice the accuracy of diagnosis, as demonstrated by the comparable performance of CNNs to that of human specialists [15].

Fremond et al.’s study places great importance on the accuracy, as seen by the reported AUROC values, which varied from 0.874 to 0.876 for both macro and class-wise analysis [16]. The values demonstrate a significant degree of accuracy in the model's capacity to accurately categorize instances of endometrial cancer. Precision of this magnitude is crucial in oncology, as accurately categorizing cancer subtypes can significantly impact therapy choices and patient results. The im4MEC model's capacity to precisely classify endometrial cancer into genetic subgroups demonstrates the promise of AI-driven technologies to improve diagnosis accuracy beyond the limitations of conventional approaches. Fremond et al.’s study highlights the capacity of deep learning models to enhance the efficiency of diagnostic procedures [16]. By employing whole-slide photos and sophisticated AI algorithms, the analysis of intricate pathological data may be conducted swiftly and comprehensively. This process, which formerly required a substantial amount of time when performed manually by pathologists, is now expedited. The ability to handle and analyze massive datasets efficiently saves time and decreases the stress on healthcare personnel, potentially enhancing the speed of diagnostic processes in clinical settings [16]. In addition, although the study did not explicitly measure the sensitivity of the im4MEC model, the high accuracy and AUROC scores indirectly indicate a high level of sensitivity. In medical diagnostics, especially in the context of cancer detection, having a high sensitivity is crucial to minimize the occurrence of false negative results. It is essential to accurately identify individuals with different subtypes of endometrial cancer to ensure they receive the most suitable and prompt treatment [16].

The most notable feature of the study, Hart et al., is the exceptional precision attained by the machine learning models, particularly the Random Forest and Neural Network models, which yielded testing Area Under the Curve (AUC) scores of 0.96 and 0.91, respectively [17]. These models' scores are markedly higher than the prior risk models, which varied between 0.68 and 0.77. Precision is paramount in cancer screening, as accurately identifying at-risk persons can significantly impact early intervention and treatment planning. By attaining exceptional precision, these models can substantially diminish overlooked diagnoses and incorrect categorizations that may result in delayed or unsuitable therapies [17]. The report emphasizes a significant transition from conventional risk assessment approaches to a more efficient and data-driven approach. Using the Prostate Lung Colorectal and Ovarian Cancer Screening Trial (PLCO) dataset, the machine learning models successfully handled large quantities of personal health data efficiently without requiring genetic imaging biomarkers or invasive procedures [17]. This risk stratification method, which is both non-invasive and cost-effective, offers a substantial enhancement in efficiency [17]. It increases the speed and capacity of data processing and alleviates the strain on healthcare systems and experts [17]. Moreover, the study's evaluation of machine learning models compared to human specialists in categorizing the risk of endometrial cancer emphasizes the dominance of AI in this field. The models demonstrated superior accuracy to human experts and significantly decreased false favorable rates. This suggests that AI can provide more dependable and accurate forecasts of risks, hence improving the overall effectiveness of the screening process [17].

Guerriero et al. examined and compared several machine learning methods, such as k-nearest neighbors (k-NN), Naive Bayes, Neural Networks, Support Vector Machine (SVM), Decision Tree, Random Forest, and Logistic Regression [18]. The models underwent training and testing using a dataset derived from a previously conducted study on intestinal endometriosis, which included 333 patients. The wide array of algorithms offers a thorough understanding of the capabilities and constraints of each model in a clinical environment. The neural network model had the highest accuracy level among the tested algorithms, with a precision of 0.73. The model's accuracy rate of 73% demonstrates its successful identification of instances, a notable accomplishment given the intricate nature of diagnosing endometriosis [18]. The model shows a balanced trade-off between sensitivity and specificity, resulting in a minimized occurrence of false positives and false negatives. The performance of these models, notably the Neural Network, is particularly remarkable compared to the conventional logistic model employed in the medical literature. The application of AI in this particular situation showcases the capacity of machine learning to enhance or even exceed traditional diagnostic techniques, offering more precise, effective, and non-intrusive alternatives for identifying endometriosis [18]. The outcomes of this study could have profound ramifications for the future of medical diagnosis. Through AI and machine learning, healthcare professionals can boost the precision of diagnoses, diminish the duration and expenses linked to conventional diagnostic techniques, and ultimately augment patient care [18]. The utilization of AI in this domain exhibits the potential for developing customized and efficient treatment strategies designed to cater to particular patients' distinct requirements and circumstances [18]. The reviewed research consistently shows that AI models, particularly convolutional neural networks (CNNs) and deep learning algorithms, are highly effective and accurate in diagnosing endometrial cancer and related diseases. Fell et al. and Takahashi et al. emphasize the capacity of AI to enhance the precision of pathology diagnoses [13,14]. CNNs have proven to be highly accurate in identifying endometrial biopsy whole slide images (WSIs), and deep neural network models have shown potential in diagnosing endometrial cancer. Additionally, these models can prioritize pathologist evaluation. This has the potential to result in expedited and more effective cancer diagnostics, thereby enhancing patient outcomes.

Urushibara et al. expand upon this concept in radiology, showcasing the accuracy of CNNs in identifying endometrial cancer using MRI. CNNs' high AUC ratings demonstrate their effectiveness in automating the interpretation of MRI scans, indicating a substantial decrease in diagnostic time compared to manual analysis performed by radiologists. CNNs' efficiency does not sacrifice accuracy, as their performance closely aligns with human experts [15].

Fremond et al. and Hart et al. emphasize the accuracy and effectiveness of AI models in oncology. The im4MEC model's capacity to categorize endometrial cancer into genetic subcategories and the notable AUC values attained by machine learning models in risk stratification illustrate the potential of AI to augment diagnostic precision and effectiveness [16,17]. These models accelerate the diagnosis process and alleviate the burden on healthcare professionals, potentially enhancing the efficiency of diagnostic procedures in clinical settings. Guerriero et al. conducted a comparative investigation of different machine learning algorithms, highlighting the neural network model's superior accuracy in diagnosing endometriosis [18]. This study demonstrates the potential of artificial intelligence (AI) to improve or exceed traditional diagnostic methods, providing more accurate, efficient, and non-invasive alternatives. Overall, the examined research suggests a significant change in medical diagnostics for AI-assisted approaches. AI's exceptional precision, effectiveness, and capacity to process extensive datasets make it a revolutionary instrument in cancer diagnosis. These developments offer improved diagnostic accuracy and the potential to decrease the time and resources needed for diagnosis, thereby relieving the strain on healthcare systems. Moreover, the capacity of AI models to distinguish between malignant and non-cancerous diseases, as well as their potential to assess the likelihood of developing certain conditions and categorize them into genetic groupings, highlights their crucial significance in personalized medicine. AI integration in medical diagnostics can enhance patient care and outcomes in oncology by enabling more personalized and efficient treatment regimens.

Quality Assessment

**Figure 2:**
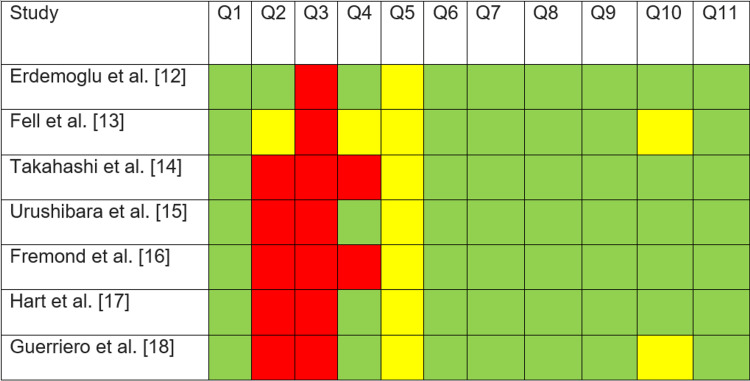
Quality assessment of articles according to the CASP checklist. Green: Yes; Yellow: Not clear; Red: No. CASP: Critical Appraisal Skills Programme Q 1. Did the study address a focused issue? Q 2. Was the assignment of patients to treatments randomized? Q 3. Were patients, health workers, and study personnel blinded? Q 4. Were the groups similar at the start of the study? Q 5. Aside from the experimental intervention, were the groups treated equally? Q 6. Were all the patients who entered the study correctly accounted for at its conclusion? Q 7. How large was the treatment effect? Q 8. How precise was the estimate of the treatment effect? Q 9. Can the results be applied in your context? (or to the local population?) Q 10. Were all clinically significant outcomes considered? Q 11. Are the benefits worth the harms and costs?

Figure 2 illustrates how the CASP checklist was incorporated to evaluate the articles' quality. Q2 [13] was not specified, considering there was no article on randomized control trials. All of the studies address a focused issue. For clinically significant outcomes, just one study [18] received a yellow because their data source was not disclosed, whereas all others had critical clinical outcomes. The overall quality of all the papers was good.

## Conclusions

This study critically evaluated the accuracy and effectiveness of AI-assisted endometrial cancer detection methods. A comprehensive analysis of multiple studies indicates that artificial intelligence can play a noteworthy and promising role in improving oncology diagnosis, explicitly identifying and categorizing endometrial cancer. The aggregate findings from every study point to a consistent advancement toward the successful application of AI in medical diagnostics. Convolutional neural networks (CNNs), in particular, are deep learning algorithms that have demonstrated exceptionally high diagnostic accuracy for endometrial cancer. It has been shown that these technologies are just as capable as human professionals at performing diagnostic duties. AI has advanced significantly in the processing and comprehending complicated medical data, making it possible to diagnose patients more quickly and accurately than ever before. Additionally, these tests have shown promising results for AI in prioritizing pathologist evaluations, which may lead to an earlier cancer diagnosis and better patient outcomes because AI is particularly good at classifying different types of cancer and differentiating benign from malignant cases. This utmost accuracy in diagnosis is critical in the field of oncology due to its substantial influence on patient prognosis and the choice of treatment.
